# Research on Golay-coded excitation in real-time imaging of high frequency ultrasound biomicroscopy

**DOI:** 10.1038/s41598-020-80406-x

**Published:** 2021-01-20

**Authors:** Xiaochun Wang, Jun Yang, Jianjun Ji, Yusheng Zhang, Sheng Zhou

**Affiliations:** Institute of Biomedical Engineering, Chinese Academy of Medical Sciences and Peking Union Medical College, Tianjin, 300192 People’s Republic of China

**Keywords:** Biomedical engineering, Electrical and electronic engineering

## Abstract

High frequency ultrasonic imaging provides clinicians with high-resolution diagnostic images and more accurate measurement results. The technique is now widely used in ophthalmology, dermatology, and small animal imaging. However, since ultrasonic attenuation in tissue increases rapidly with increasing frequency, the depth of detection of high frequency ultrasound in tissue is limited to a few millimeters. In this paper, a novel method of using Golay-coded excitation as a replacement for conventional single-pulse excitation in high frequency ultrasound biomicroscopy was proposed, and real-time imaging was realized. While maintaining the transmission voltage and image resolution unchanged, the detection depth can be effectively improved. The ultrasonic transmission frequency is 30 MHz and the transmission voltage is ± 60 V p-p. In this study, 4-bit, 8-bit, and 16-bit coding sequences and decoding compression were used. To verify the effectiveness of the coding sequence in real-time imaging of ultrasound biomicroscopy, we designed a 10-μm diameter line target echo experiment, an ultrasound phantom experiment, and an in vitro porcine eye experiment. The experimental results show that the code/decode method of signal processing can not only maintain a resolution consistent with that of single-pulse transmission, but can also improve the detection depth and signal-to-noise ratio.

## Introduction

Compared to conventional medical ultrasound diagnostic technology, ultrasonic imaging at high frequencies of 30–100 MHz can provide clinicians with clearer diagnostic images and more accurate measurement results^1^. High frequency medical ultrasonic imaging has been widely used in the examination of glaucoma^2^ and diseases in the anterior segment tissues of the eye, such as cornea, ciliary, and lens^3–5^, as well as human epidermis^6^, hair follicles, subcutaneous tissue, superficial blood vessels^7^, and on experimental small animals^8,9^. It thus has important research and diagnostic value. At the same time, because acoustic energy at high frequency attenuates rapidly in human tissue^10^, the depth of detection of high frequency ultrasound biomicroscopy in tissues is limited to a few millimeters because the echo information in deep tissue is lost. This high attenuation greatly affects its application value in clinical diagnosis.

Mamou and Ketterling et al.^11–17^ demonstrated that the transmission of chirp-coded excitation from a circular array can improve the image quality of high frequency ultrasonic images, and they successfully validated this technique in small animal imaging. Members of the Kirk Shung research team used Barker code^18^ and chirp code^19–23^ to design and implement a high-frequency coded excitation and reception system that initially achieved imaging of small animal hearts. However, among today's commercially available instruments, ultrasonic imaging equipment using coded excitation technology is only concentrated in the usual frequency band of ordinary ultrasound. The research results described above are still in the laboratory stage, and real-time imaging using chirp-coded frequencies has not been realized due to the demanding requirements on the transmitting system.

Therefore, in this study, we introduce digital Golay-coded excitation instead of the conventional single-pulse excitation in high frequency ultrasound biomicroscopy imaging technology. In addition to realizing real-time imaging and improving the depth of detection and signal-to-noise ratio, the method can also maintain the same transmission voltage and image resolution. And now, this research achievement has been transformed into a commercial ultrasound biomicroscope.

## Results

### Computer simulation results

**Figure 1:**
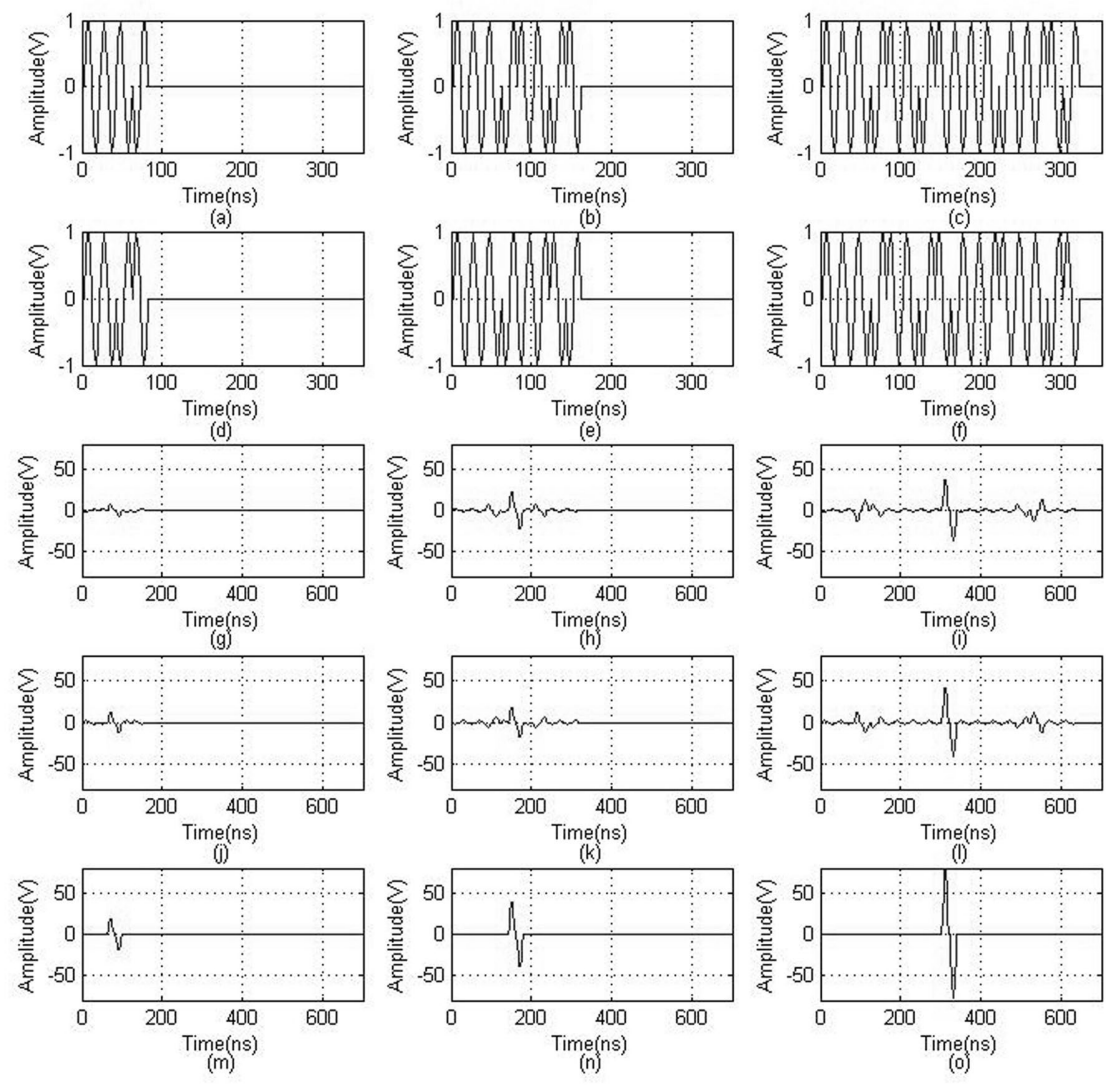
Computer simulation results for Golay coding and decoding. (**a**–**c**) are respectively the 4-bit, 8-bit, and 16-bit Golay sequence A codes for encoding. (**d**–**f**) are respectively the 4-bit, 8-bit, and 16-bit Golay sequence B codes for encoding. (**g**–**i**) are respectively the 4-bit, 8-bit, and 16-bit Golay sequence A codes for decoding. (**j**–**l**) are respectively the 4-bit, 8-bit, and 16-bit Golay sequence B codes for decoding. (**m**–**o**) are respectively the 4-bit, 8-bit, and 16-bit Golay decoding results.

As shown in Fig. 1a–c are respectively the 4-bit, 8-bit, and 16-bit Golay sequence A codes, (d–f) are respectively the 4-bit, 8-bit, and 16-bit Golay sequence B encoding codes, (g–i) are respectively the 4-bit, 8-bit, and 16-bit Golay sequence A decoding codes, (j–l) are respectively the 4-bit, 8-bit, and 16-bit Golay sequence B decoding codes, and (m–o) are respectively the 4-bit, 8-bit, and 16-bit Golay decoding results.

As can be seen from Fig. 1a–f, the width of the signal increased as the number of coded pulses increased. Compared to the conventional single-pulse excitation method, this method increases the time for carrying the sound source information and also increases the average transmission power of the ultrasonic signal. It can be seen from Fig. 1 (m–o) that the decoded echo can completely eliminate the side lobes and effectively increase the signal amplitude while ensuring the same resolution as the single-pulse transmission and reception.

### Experimental results of wire target echoes

**Figure 2:**
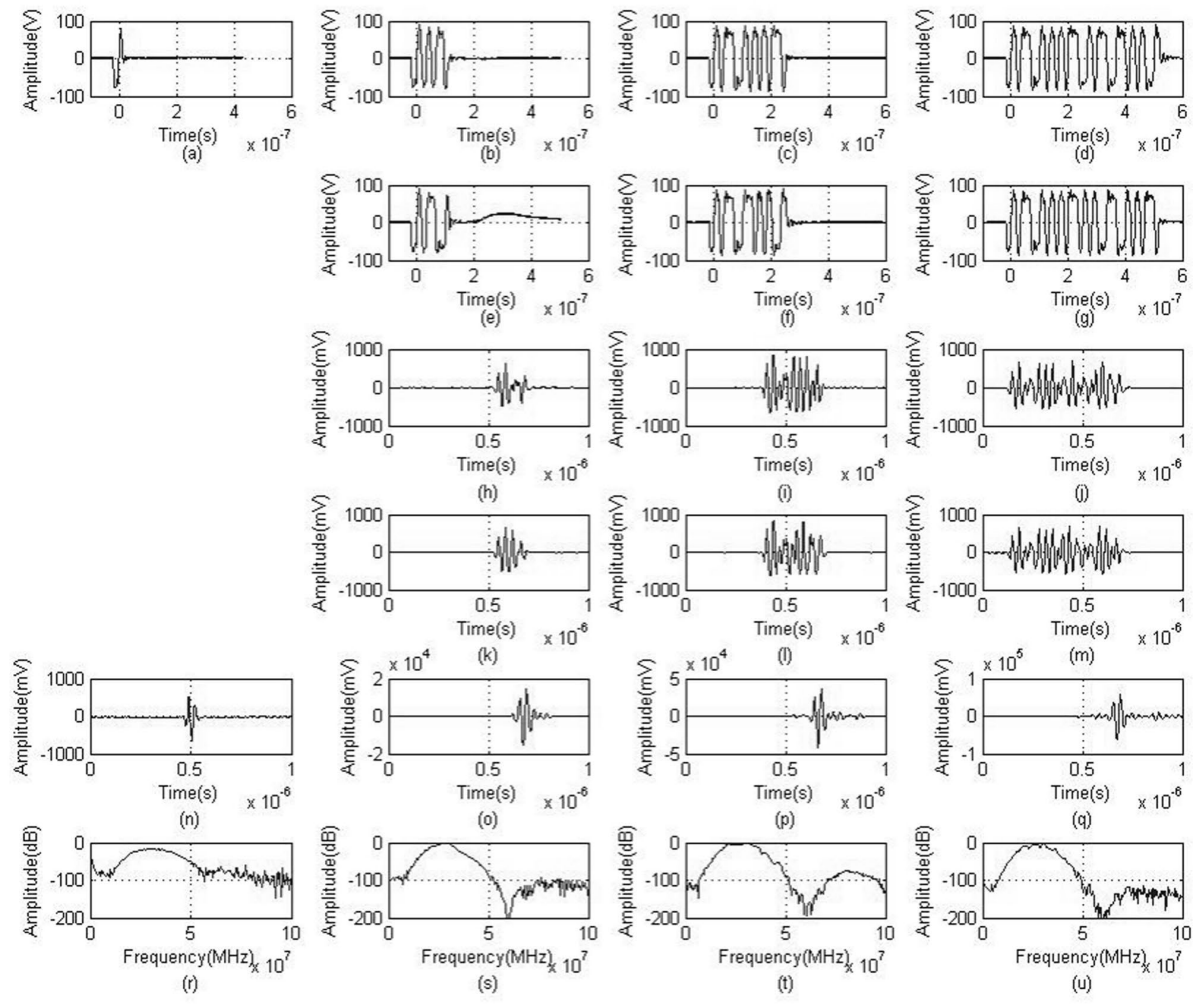
Experimental results of wire target echoes. (**a**) Single-pulse transmission waveforms. (**b**–**d**) are respectively the 4-bit, 8-bit, and 16-bit Golay sequence A coded transmission waveforms. (**e**–**g**) are respectively the 4-bit, 8-bit, and 16-bit Golay sequence B coded transmission waveforms. (**h**–**j**) are respectively the 4-bit, 8-bit, and 16-bit Golay sequence A coded return echoes. (**k**–**m**) are respectively the 4-bit, 8-bit, and 16-bit Golay sequence B coded return echoes. (**n**) is the return echo of a single pulse. (**o**–**q**) are respectively the 4-bit, 8-bit, and 16-bit Golay sequence decoded return echoes. (**r**–**u**) are respectively the frequency spectra of the single-pulse echo and the 4-bit, 8-bit, and 16-bit Golay sequence echoes.

As shown in Fig. 2a is the actual transmitted waveform of a single pulse, (b–d) are respectively the 4-bit, 8-bit, and 16-bit Golay sequence A encoded transmission waveforms, (e–g) are respectively the 4-bit, 8-bit, and 16-bit Golay sequence B encoded transmission waveforms, (h–j) are respectively the 4-bit, 8-bit, and 16-bit Golay sequence A encoded return echoes, (k–m) are respectively the 4-bit, 8-bit, and 16-bit Golay sequence B encoded return echoes, (n) is the received echo of a single pulse, (o–q) are respectively the 4-bit, 8-bit, and 16-bit Golay sequence decoded echoes, and (r–u) are respectively the echo spectra of a single pulse and 4-bit, 8-bit, and 16-bit Golay sequences.

It can be seen from the decoded echoes in (o–q) that the coded excitation can effectively increase the amplitude of the echo signal, but it cannot completely eliminate the side lobes as the decoded echo had done in the previous computer simulation. From the (r–u) echo spectra, we notice that the peak frequencies are all around 30 MHz. With coded excitation, the frequency of the decoded echo does not change.

**Figure 3:**
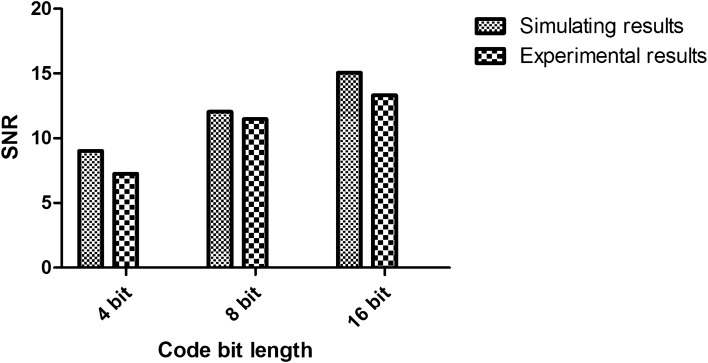
SNR improvement of the echo signal under Golay code excitations.

We then analyzed the signal-to-noise ratio. The signal-to-noise ratios of a single pulse excitation and Golay sequence excitation are shown in Fig. 3. Under the Golay sequence-coded excitation, the code lengths were respectively 4 bits, 8 bits, and 16 bits. The signal-to-noise ratio increased linearly with increasing length of the excitation code. From the computer simulation results, the theoretical values of the increased signal-to-noise ratio are 9.03 dB, 12.04 dB, and 15.05 dB, consistent with the results deduced from Eq. (2). The signal-to-noise ratio gains analyzed based on the actual signals were 7.26 dB, 11.47 dB, and 13.32 dB. A comparison of the two sets of experimental results shows that the actual signal was basically in agreement with the simulated signal. The signal-to-noise ratio of the actual signal was lower than that of the simulated signal, perhaps because the measured signal was affected by the transducer bandwidth and noise. The deviations between the actual signal and the simulated signal were respectively 19.6%, 4.7%, and 11.5%.

**Figure 4:**
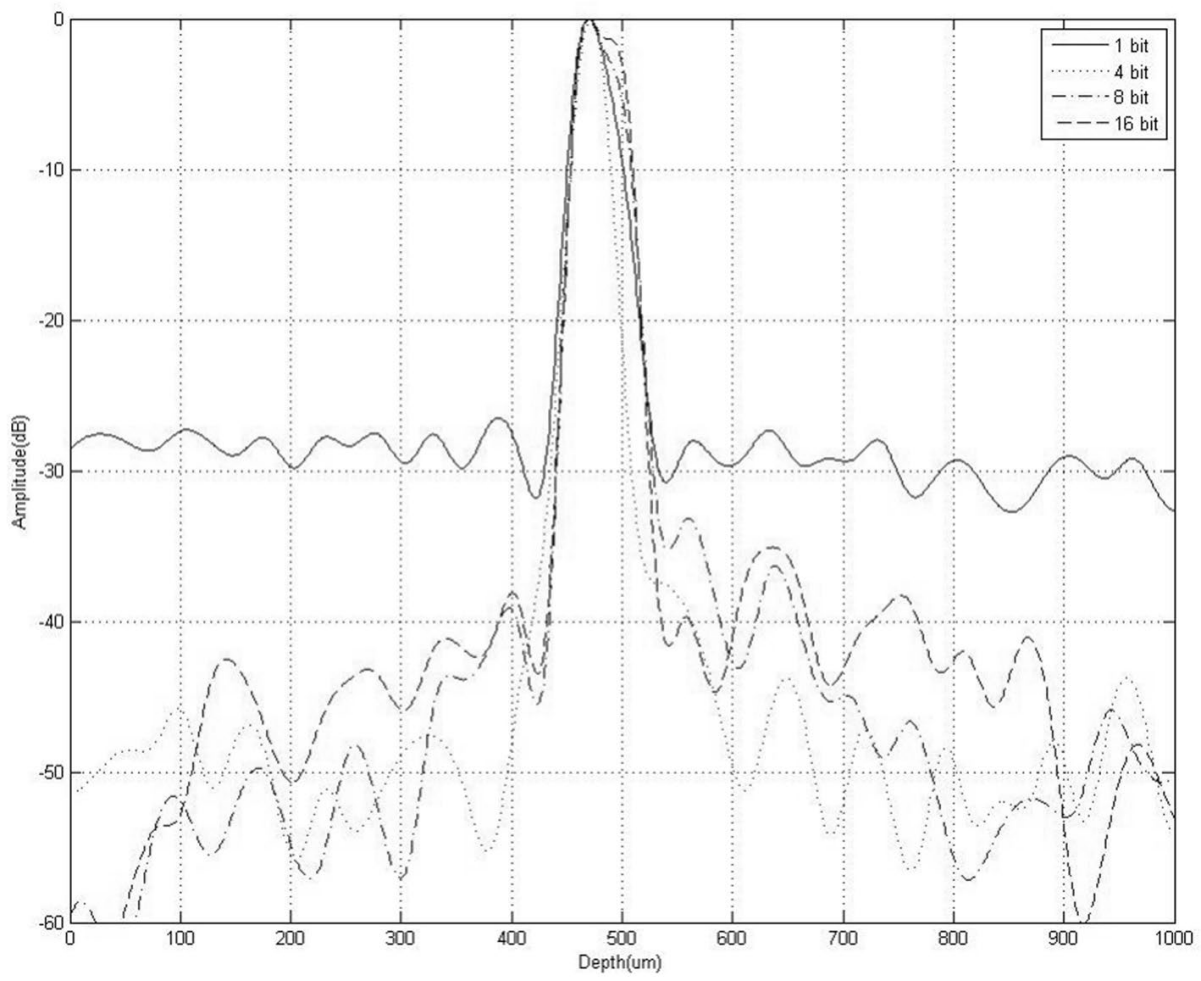
Envelope of decoded echo signals.

Figure 4 shows the envelope of the decoded echo signal that exhibits the characteristics of the side lobes. When the conventional single-pulse transmission was used, the level of the side lobe of the echo was around − 30 dB. When the coded transmission was used, the side lobe amplitude of the echo was significantly reduced. It can be concluded from the figure that when single-pulse transmission and 4-bit, 8-bit, and 16-bit Golay sequence-coded transmission are used, the − 6 dB axial resolutions of the decoded echo signal are respectively 46 μm, 51 μm, 50 μm, and 52 μm without any appreciable broadening.

### Resolution test results

**Figure 5:**
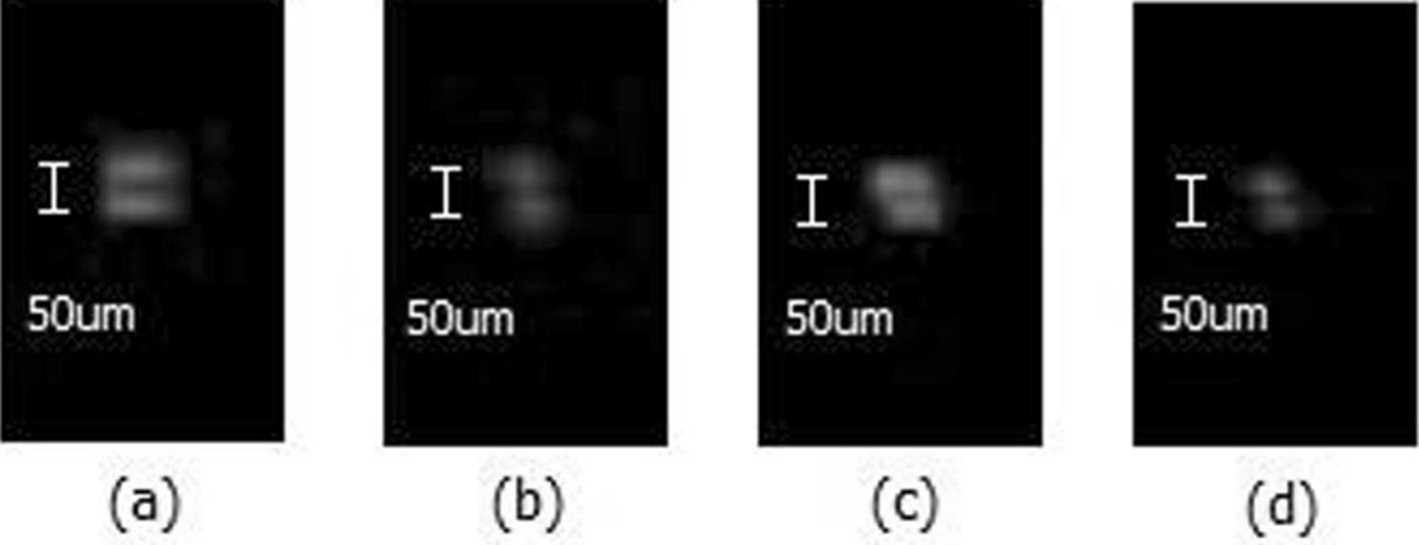
The results of resolution test imaging: (**a**) Single-pulse transmission. (**b**) 4-bit Golay-coded transmission. (**c**) 8-bit Golay-coded transmission. (**d**) 16-bit Golay-coded transmission.

Figure 5 shows the test results of the axial resolution of an acoustic microscope. Figure 5a shows the results of single-pulse transmission imaging, and Fig. 5b,c,d are respectively the results for 4-bit, 8-bit, and 16-bit Golay-coded excitation imaging. The gains of the images were all 15 dB. The experimental results show that when we scan the two tungsten wire targets after adjusting the display parameters, the probe, and the target all to optimal conditions, the real-time scanned image using coded excitation and decoding compression can still clearly resolve the two wires. Therefore, the resolution of the system can reach 50 μm, which satisfies the clinical requirements for axial resolution of a high frequency acoustic microscope.

### Experiment results on phantom imaging

**Figure 6:**
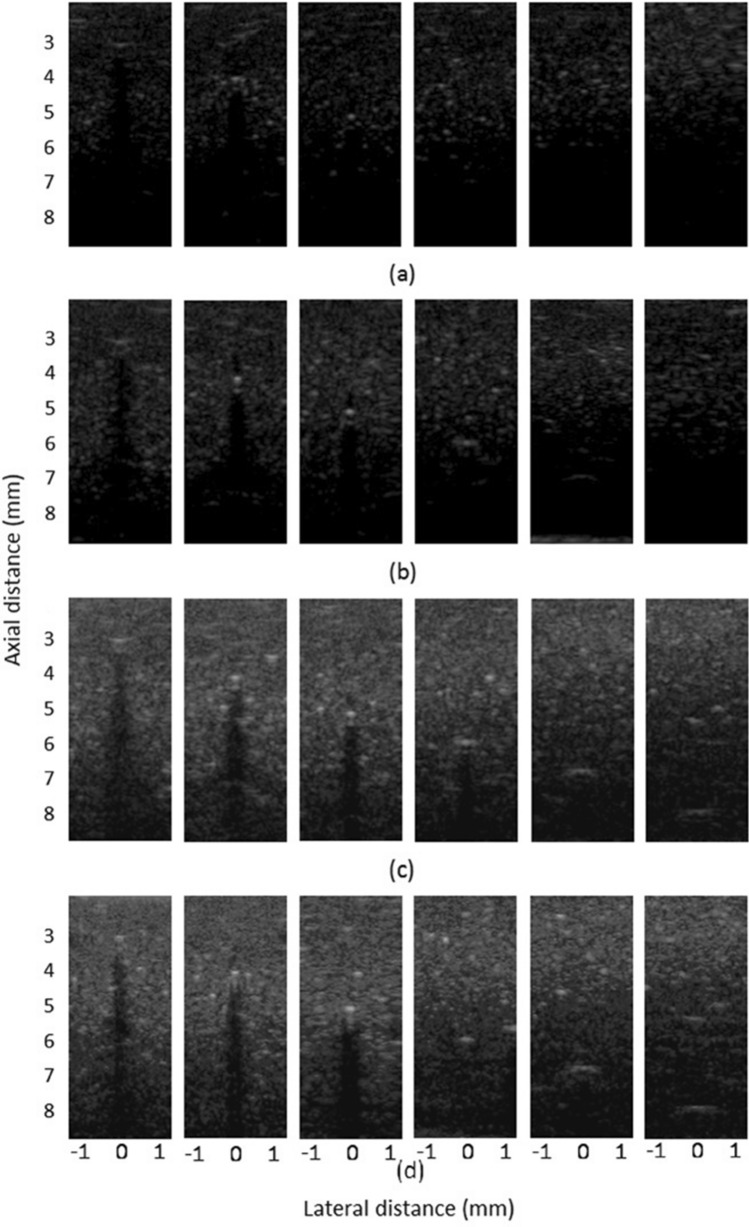
Tissue-mimicking ultrasound phantom imaging results: (**a**) Single-pulse real-time scan image result. (**b**–**d**) are respectively the real-time imaging results for 4-bit, 8-bit, and 16-bit coded excitation after decoding compression.

Figure 6 shows the imaging results of a tissue-mimicking ultrasound phantom. The image gain was 75 dB. Among them, (a) is the real-time scan image result using a single pulse, and (b–d) are the real-time imaging results obtained from 4-bit, 8-bit, and 16-bit coded excitation and decoding compression, respectively. It can be seen from the results that the depth of detection increases with an increasing number of coded bits while keeping the axial and lateral resolution constant. At the same time, as the number of coded bits increases, the ability to detect small signals is also gradually improved.

### In vitro porcine eyeball experimental results

**Figure 7:**
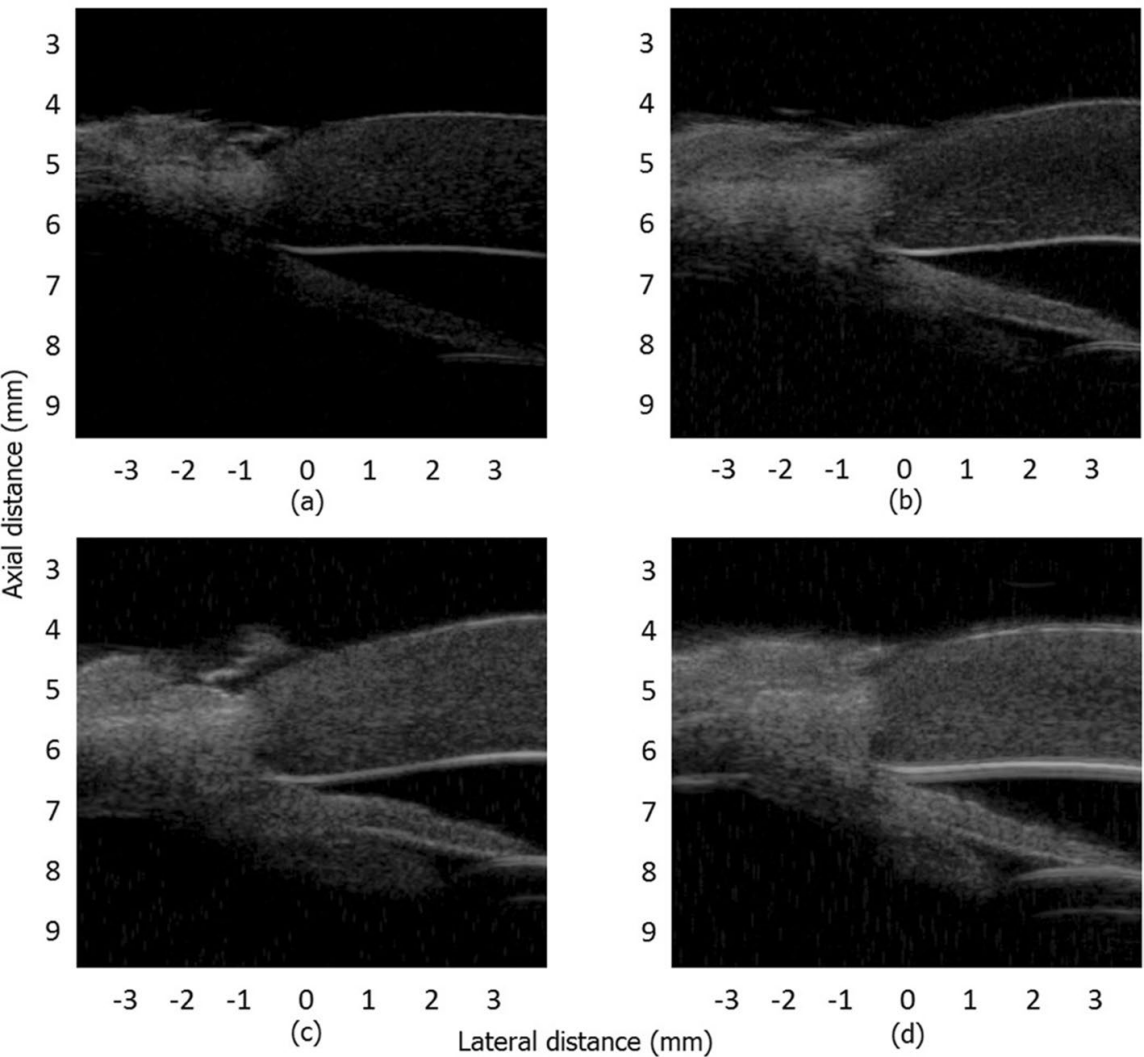
Imaging results of a corner segment of an in vitro porcine eyeball. (**a**) Real-time scan image using a single pulse. (**b**–**d**) Real-time imaging results using 4-bit, 8-bit, and 16-bit coded excitation and decoding compression, respectively.

Figure 7 shows the real-time imaging results of an in vitro porcine eye. For all the images, the dynamic range was set at 80 dB. Figure 7a–d are respectively the imaging results for a single pulse, 4-bit Golay-coded excitation, 8-bit Golay-coded excitation, and 16-bit Golay-coded excitation. As can be seen from the figure, the image using coded excitation is overall brighter than that of a single-pulse excitation, indicating that the echo signal with coded excitation is much greater than the echo signal from a single pulse. The results also show that, as the number of coded bits increases, the information of the image on the whole is enhanced. This not only effectively improves the depth of detection, but also improves the capability for detecting small signals and significantly improves the signal-to-noise ratio as well. For example, the iris is barely visible in the first frame, but by the time you get to the last frame the iris, ciliary body, maybe zonules, and more layers of the cornea all become visible.

## Discussion

In this paper, we introduce a method for replacing conventional single-pulse excitation with digital Golay-coded excitation in high frequency ultrasound biomicroscopy imaging. The detection depth and signal-to-noise ratio are effectively improved, while the transmission voltage and image resolution remain the same as those in single-pulse excitation.

In our experiments, the coded ultrasonic transmission pulses were generated by a FPGA, and the high frequency ultrasonic transmission module was driven to emit an ultrasonic excitation with an amplitude of ± 60 V p-p for the ultrasonic transducer. When a single pulse is transmitted, the transmission frequency can be adjusted to 50 MHz. In transmitting coded pulses, when the code changes from "+ 1" to "− 1" or from "− 1" to "+ 1", waveform distortion can occur at the connection point of the two waveforms^24^. Waveform distortion is caused by the limited bandwidth of the transmitter module and transducer and brings other undesired frequency components into the signal. Therefore, if the excitation frequency of the coded transmission is to be increased, it is necessary to select a suitable transducer and a transmission module that are conducive to the reduction of waveform distortion.

The signal-to-noise ratio of the actual echo signal in our experiment was basically consistent with but lower than the signal-to-noise ratio of the computer simulation signal. The reason may be that the signal transmission and measurement are affected by the bandwidth and noise of the transducer. In future investigations, the instantaneous frequency characteristics of the encoded impulse response should be matched to that of the transducer in order to reduce the power loss in the transducer and to increase the signal-to-noise ratio^25^.

Table 1 shows the comparison between our experimental results and other code results from the citation. It can be seen that for different codes and different center frequencies, the − 6 dB axial resolution and SNR will be improved to a certain extent. Because of the bandwidth limitation of the anterior stage transmission module and the realizability of real-time imaging for FPGA algorithm, we only studied a Golay code sequence in this research. Computer simulation results show that the Golay complementary sequence code can completely eliminate side lobes, but the two transmissions of the Golay complementary sequence may cause position offset of the tissue specimen in the two transducer movements, leading to side lobe artifacts in the image^26^. These artifacts can be particularly prominent in the border region of the tissue. Therefore, other phase coding modes could be introduced in future research, such as M-sequence^27^ and chirp code^28^. At the same time, new pulse compression methods such as mismatched filter^29^, Wiener filter^25^, and others can be introduced in further research to reduce the influence of side lobes on the compressed echo signals and to improve the signal-to-noise ratio and image quality.

**Table 1:** Comparison between our experimental results and other code results from the citation.

Excitation signal	Center frequency (MHz)	− 6 dB axial resolution (um)	SNR improvement (dB)
Chirps	70	28.8	16.4
Chirps	46	35	15
Chirps	40	43	12.5
Chirps	31	44	14
Chirps	17	130	11.9
13-bit Barker	40	64	11.14
40MPS	40	40	16.02
4-bit Golay	30	51	7.26
8-bit Golay	30	50	11.47
8-bit Golay	30	52	13.32

In this study, we propose a method that replaces the conventional single-pulse excitation with digital Golay-coded excitation in high frequency ultrasound biomicroscopy imaging. Both the computer simulation experiment and real-time imaging experiment showed that encoding excitation and decoding compression methods can effectively improve the detection depth and the signal-to-noise ratio of the image while keeping the transmission voltage and image resolution unchanged.

## Materials and methods

Coded ultrasonic imaging has become a popular research topic in the field of medical ultrasound diagnostic imaging in recent years. Compared to conventional single-pulse excitation, the coded excitation technique transmits a long coded pulse sequence and receives an echo that is also a long pulse sequence; the pulses are compressed by a matched filter or unmatched filter to obtain a spatial resolution close to that of the single-pulse excitation. Coded excitation technology can significantly increase the average transmitted power, increase the penetrating power, and improve the signal-to-noise ratio without increasing the peak transmitted power^29^. Practice has proven that the use of coded excitation technology in ultrasound imaging can increase the scanning depth and improve the signal-to-noise ratio^27^.

The Golay code, also known as a Golay complementary sequence pair, is defined as a pair of equal length, finite sequences of two elements. And for any given interval, the number of identical element pairs in one sequence is equal to the number of different element pairs in the other sequence. The condition that a pair of bidirectional sequences A and B of length N are Golay complementary sequences holds true if and only if the following formula holds:

$$ {\text{a}}\left( {\text{n}} \right)*{\text{a}}\left( { - {\text{n}}} \right) + {\text{b}}\left( {\text{n}} \right)*{\text{b}}\left( { - {\text{n}}} \right) = {\text{2N}} \left( {\text{n}} \right) $$

A Golay complementary sequence pair can be constructed recursively from other complementary sequence pairs. Given a Golay pair {A, B}, another Golay pair of twice the length can be generated by {AB, A(− B)}. This recursion can start with a Golay pair of length 2 for A = [1, 1] and B = [1, − 1]. The Golay sequence pairs used in the experiment here are shown in Table 2.

**Table 2:** Golay-code with different bit lengths.

Bit length	Golay-code (A)	Golay-code (B)
4	+ 1, + 1, + 1, − 1	+ 1, + 1, − 1, + 1
8	+ 1, + 1, + 1, − 1, + 1, + 1, − 1, + 1	+ 1, + 1, + 1, − 1, − 1, − 1, + 1, − 1
16	+ 1, + 1, + 1, − 1, + 1, + 1, − 1, + 1, + 1, + 1, + 1, − 1, − 1, − 1, + 1, − 1	+ 1, + 1, + 1, − 1, + 1, + 1, − 1, + 1, − 1, − 1, − 1, + 1, + 1, + 1, − 1, + 1

**Figure 8:**
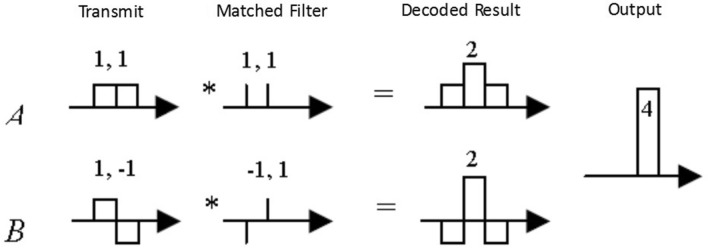
Pulse compression of the Golay code.

The way that Golay sequence pairs are used in imaging is to make two transmissions for each focus point. After the first transmission of Golay code A, the echo signal is filtered by the corresponding decoding filter A (that is, as a correlation operation) and stored in the buffer memory. This is followed by the transmission of Golay code B, and the echo signal is filtered by the corresponding decoding filter B. The decoding process is completed by adding the two filtered output waveforms according to the above complementary conditions, as shown in Fig. 8.

Theoretically, the coded transmission of the Golay complementary sequence pair can completely eliminate the side lobes while keeping the width of the main lobe constant. However, due to the motion of the tissue between the two transmissions, the theoretical effect cannot be obtained in practical application. In addition, the use of Golay code will reduce the frame rate of the image by half^30^.

Studies have shown that, by using coded excitation, the signal-to-noise ratio can be raised by a maximum of 15–20 dB theoretically. For a binary code of length N, the gain in signal-to-noise ratio by using matched filter pulse compression is:

1 $$ {\text{SNRgain}} = {1}0{\text{logN}} $$

Therefore, for a coded excitation using a Golay complementary sequence pair of length N, two transmissions are required, and the corresponding signal-to-noise ratio gain^18,31^ is:

2 $$ {\text{SNRgain}} = {1}0{\text{log2N}} $$

### Computer simulation experiment

In order to assess the feasibility of using a Golay code sequence in ultrasound bio-microscopic imaging, a computer simulation experiment was performed. Since the sampling frequency for an ultrasonic echo is generally 8 times the frequency of the ultrasonic echo, a sinusoidal waveform [0, − 7, − 10, − 7, 0, 7, 10, 7] is used to represent − 1 and [0, 7, 10, 7, 0, − 7, − 10, − 7] is used to represent + 1 in the encoding process. Comparisons were made using Golay sequences of 4-bits, 8-bits, and 16-bits, and a matched filter decoding method was adopted in the decoding process. Finally, the improvements in signal-to-noise ratio by Golay sequence codes of different bits were compared (see the Computer Simulation Results).

### Echo experiment using a tungsten wire target

**Figure 9:**
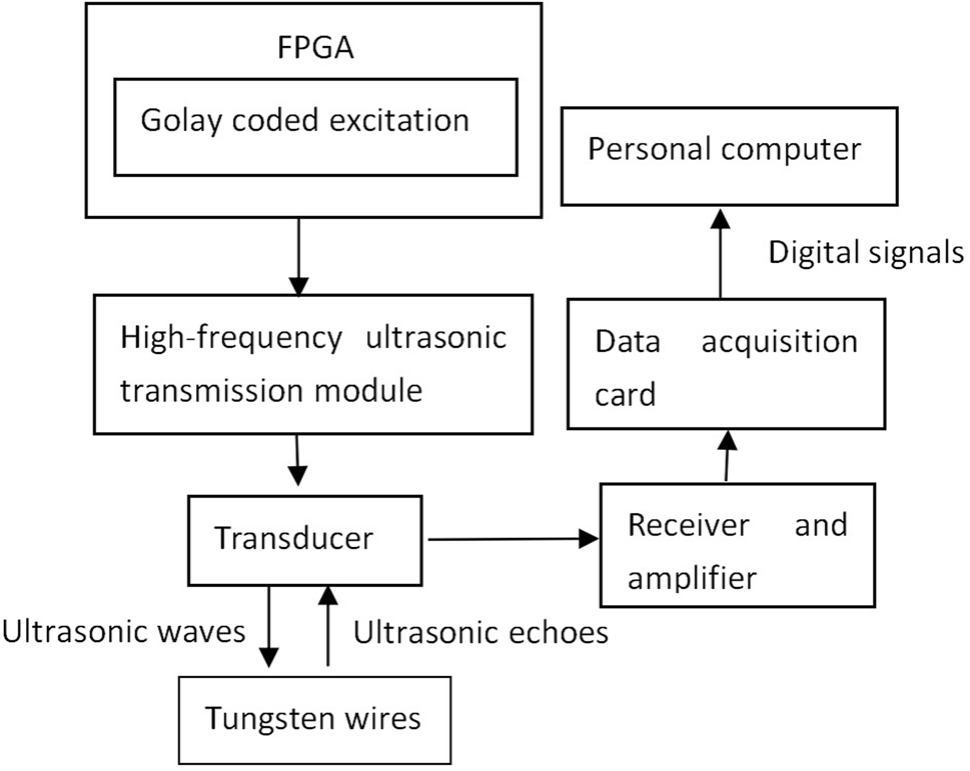
Block diagram of the echo experimental system.

In order to verify the effectiveness of the Golay sequence code in high frequency acoustic microscope imaging, we designed an echo experiment using a tungsten wire target. A schematic diagram of the experiment is shown in Fig. 9.

A tungsten wire target of 10 μm diameter and placed in bubble-free water was used as the experimental sample. A single element polyvinylidene fluoride (PVDF) broadband ultrasonic transducer (AT19344, MedTech, Potomac, Maryland, USA) with a center frequency of 30 MHz and a bandwidth of 55% was used. The diameter of the active element was 5 mm ± 0.5 mm. The transducer had a focal length of 9 mm ± 0.5 mm, so it was also placed in the bubble-free water at a distance of 9 mm vertically above the tungsten wire target. The ultrasonic transmissions were 4-bit, 8-bit, and 16-bit single-pulse Golay-coded excitations generated by a field programming gate array (FPGA) (EP4CE22F17C8N, Altera, San Jose, California, USA) at a frequency of 30 MHz^32^. The coded ultrasonic transmission pulses drove a high-frequency ultrasonic transmission module (TC6320, MICROCHIP, Chandler, Arizona, USA) to generate an ultrasonic excitation of ± 60 V p-p amplitude at the ultrasonic transducer. The ultrasonic transducer simultaneously transmitted ultrasonic waves to the tungsten wire target and received ultrasonic echoes from the target. The echoes were amplified by the preamplifier circuit (AD8331, ADI, Norwood, Massachusetts, USA) with a total gain of 80 dB. The echo signal was then digitized by the data acquisition card (QT1135AC-2, Queentest, Beijing, CHINA) and read to the computer for decoding and compression by MATLAB software (MathWorks Inc., Natick, MA, USA). Finally, the effects of the Golay sequence code on the actual signal-to-noise ratio and image resolution were compared.

### Real-time imaging system

**Figure 10:**
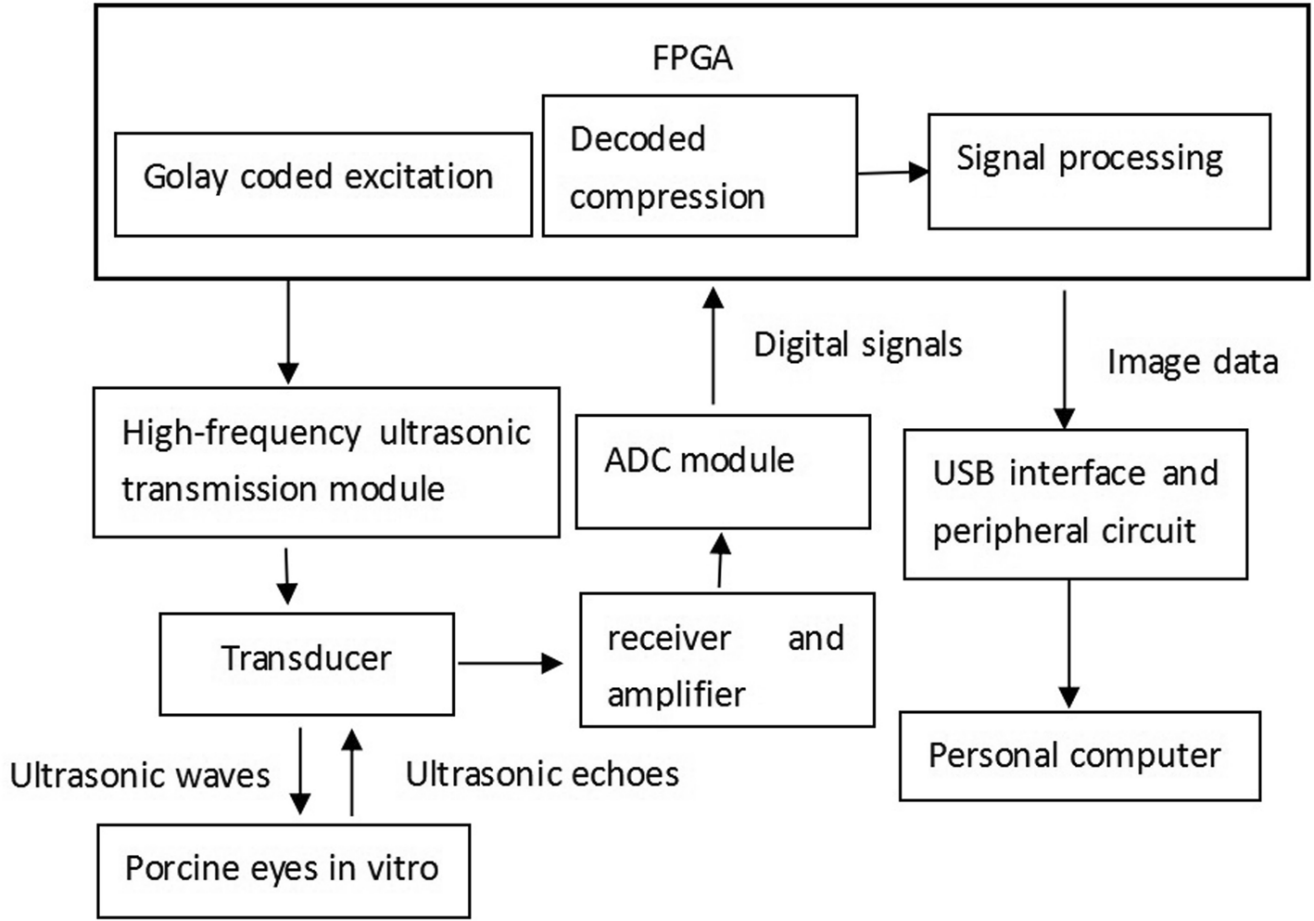
Block diagram of the real-time imaging system.

The construction of the high frequency acoustic microscope real-time imaging system is shown in Fig. 10.

The single-element transducer was fixed on a linear guide rail, and the transducer was driven by a stepping motor to do mechanical linear reciprocating scanning. Based on the echo experiment using the tungsten wire target, a tunable amplifier with an adjustable gain range of 40 dB (AD8331, ADI, Norwood, Massachusetts, USA) was added. Also, a time gain control was used according to the detection depth. The echo signal was digitized by two high-speed 14 bit analog-to-digital converters (LTC2285, ADI, Norwood, Massachusetts, USA) at a sampling frequency of 120 MHz. The two sampling clocks were 180 degrees out of phase, and after phase interpolation, an echo signal with a sampling frequency of 240 MHz was obtained. The digitally sampled echo signal was sent to the FPGA (EP4CE22F17C8N, Altera, San Jose, California, USA) after echo decoding, compression, and subsequent digital signal processing such as bandpass filtering, timegain controlling, demodulating, logarithm amplifying, twice sampling, and then transmitted to the host PC through a USB port for real-time image display.

The repetition period of the transmission pulse was 200 μs, and a set of coding pulses was transmitted every 100 μs. The first 100 μs transmitted the A code, followed by the transmission of the B code in the next 100 μs. The received echoes were separately decoded and compressed, and then superimposed to obtain the echo data of one line.

### Axial resolution test

The resolution of ultrasonic imaging was divided into lateral resolution (perpendicular to the beam direction) and axial resolution (parallel to the beam direction). Since acoustic microscope scans with a single element probe in a linear fashion, the lateral resolution is mainly determined by the transducer selected and is unaffected by the coding excitation and decoding compression. Therefore, we only need to test the axial resolution to observe the effect of the Golay-coded excitation on the resolution and to determine whether the resolution requirements of a clinical acoustic microscope are met. The definition of axial resolution is the ability to resolve two adjacent echoes in the axial direction of the ultrasonic beam. The experimental results show that the distance between the two resolvable echoes in the image was inversely proportional to the resolution of the system. The smaller the distance that can be resolved, the greater the axial resolution of the system.

**Figure 11:**
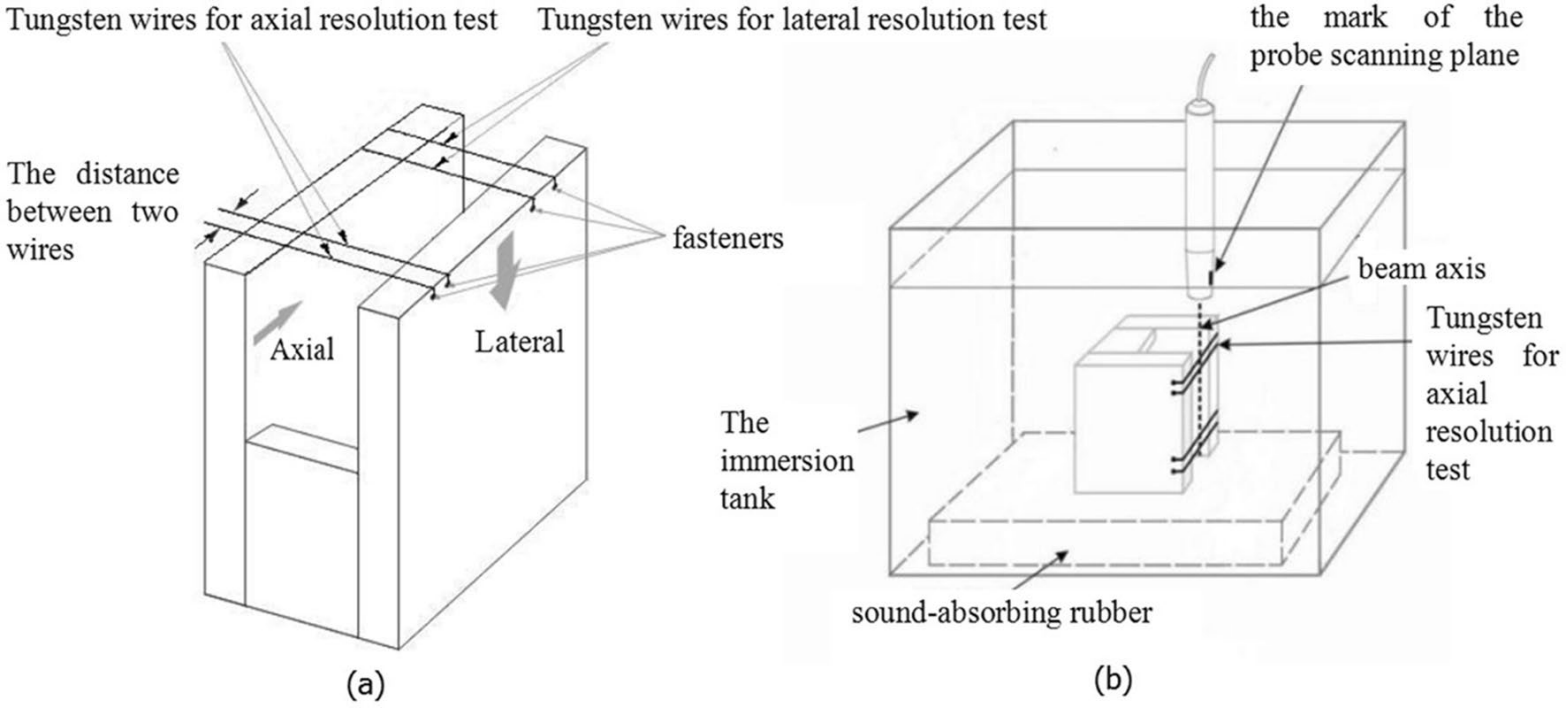
Diagram of the line target for the resolution test. (**a**) The wire target for resolution test. (**b**) Diagram of ultrasonic scanning system.

The test block used in this resolution test experiment was the wire target shown in Fig. 11a. For the tungsten wire line target, two fasteners were attached to a U-shaped piece of Plexiglas, and two 10-μm diameter tungsten wires were wrapped tightly between the two fasteners. The two wires were parallel and separated by a distance of 50 μm. The separation distance was verified with a calibration microscope. The bottom of the immersion tank was lined with sound-absorbing rubber upon which the testing line target was placed. The two target wires were aligned parallel to the propagation direction of the ultrasound; that is, the plane containing the parallel target wires was perpendicular to the surface of the probe. The immersion tank was slowly filled with degassed distilled water, the surface bubbles were removed, and the water was allowed to sit for 10 min. The hand-held UBM probe was positioned above the wire target and scanned in a plane perpendicular to the target wires as shown in Fig. 11b. The distance between the probe and the target wires was adjusted to 9 mm, and the gain, contrast, and brightness were optimized. The image of the wire target was viewed on the screen, and the axial resolution of the system reached 50 μm when the two tungsten wire targets could be clearly resolved on the image.

### Phantom experiment

Following the national requirements YY0849-2011 for industrial standards for high-frequency diagnostic ophthalmic instruments, the experiment employed a tissue-mimicking ultrasound phantom (KS107BG, Institute of Acoustics, Chinese Academy of Sciences, Beijing, China) to measure the improvement in detection depth of coded excitation. The target wires of the phantom from the acoustic window were 2 mm, 3 mm, 4 mm, 5 mm, 6 mm, 7 mm, and 8 mm, respectively. First removed the cover plate and the sponge cushion of the phantom for protection. Then poured a proper amount of distilled water into the water tank as the coupling medium between the probe and the acoustic window. The hand-held UBM probe was placed on the acoustic window and scanned in a plane perpendicular to the target wires of the phantom. Under the premise of keeping the axial and lateral resolution unchanged, the phantom was used to verify the influence of the number of bits of the coded excitation on the depth of detection^33^.

### Experiment in vitro on a porcine eye

Real-time imaging was also performed in vitro on a porcine eye. The experimental specimens were obtained at local slaughterhouses from pigs approximately 1 year old. After removing residual extraneous tissues around the eye with a scalpel, the eyeball was carefully cleaned in a saline buffer. The cleaned porcine eyeball was placed promptly in a container filled with distilled water and held in place. The distance between the probe and the eyeball was adjusted to the optimum 9 mm, and the gain, contrast, and brightness were all optimized and maintained constant throughout the experiment.
